# Hibernation with Rhythmicity in the Retina, Brain, and Plasma but Not in the Liver of Hibernating Giant Spiny Frogs (*Quasipaa spinosa*)

**DOI:** 10.3390/biology11050722

**Published:** 2022-05-09

**Authors:** Zhigang Xie, Ibrahim M. Ahmad, Lirong Zuo, Hui Wang, Dongming Li

**Affiliations:** 1 Institute of Hydrobiology, Zhejiang Academy of Agricultural Sciences, Hangzhou 310021, China; 2 Ministry of Education Key Laboratory of Molecular and Cellular Biology, Key Laboratory of Animal Physiology, Biochemistry and Molecular Biology of Hebei Province, College of Life Sciences, Hebei Normal University, Shijiazhuang 050024, China; ibgausee@yahoo.com (I.M.A.); lirong.zuo@outlook.com (L.Z.); wanghui@hebtu.edu.cn (H.W.)

**Keywords:** circadian clock genes, antioxidant enzymes, giant spiny frogs, hibernation, molecular adaptation, cold stress, hypoxia

## Abstract

**Simple Summary:**

Aquatic ectotherms experience hypoxia under water during hibernation, which enables them to move denoting some level of consciousness, unlike terrestrial hibernators. However, how aquatic ectotherms modulate their clocks and clock-controlled genes in different tissues and plasma melatonin and corticosterone in light-dark cycles under natural environments before and during hibernation, remains to be largely unexplored. To achieve these, in this study, we investigated circadian clock genes, circadian clock-controlled genes, antioxidant enzyme genes, and related hormones in giant spiny frog (*Quasipaa spinosa*). Our results demonstrated that, despite the hypometabolic state of hibernation, the retina and the brain displayed some circadian rhythms of clock and antioxidant genes, as well as melatonin, while the liver was inactive. These novel findings may contribute to an understanding of how aquatic ectotherms use their circadian system differentially to modulate their physiology in escaping hypoxia during hibernation and preparing for arousal.

**Abstract:**

Hibernation in ectotherms is well known, however, it is unclear how the circadian clock regulates endocrine and antioxidative defense systems of aquatic hibernators. Using the giant spiny frog (*Quasipaa spinosa*), we studied mRNA expression levels of (1) circadian core clock (*Bmal1*, *Clock*, *Cry1* and *Per2*), clock-controlled (*Ror-α*, *Mel-1c* and *AANAT*), and antioxidant enzyme (AOE) (*SOD1*, *SOD2*, *CAT* and *GPx*) genes in retina, brain, and liver; and (2) plasma melatonin (MT) and corticosterone (CORT) levels, over a 24-hour period at six intervals pre-hibernation and during hibernation. Our results showed that brain *Bmal1*, *Cry1*, *Per2* and *Mel-1c* were rhythmic pre-hibernation and *Clock* and *Ror-α* during hibernation. However, the retina *Bmal1*, *Clock* and *Mel-1c*, and plasma MT became rhythmic during hibernation. All brain AOEs (*SOD1*, *SOD2*, *CAT* and *GPx*) were rhythmic pre-hibernation and became non-rhythmic but upregulated, except *SOD1,* during hibernation. However, plasma CORT and liver clocks and AOEs were non-rhythmic in both periods. The mRNA expression levels of AOEs closely resembled those of *Ror-α* but not plasma MT oscillations. In the hibernating aquatic frogs, these modulations of melatonin, as well as clock and clock-controlled genes and AOEs might be fundamental for them to remain relatively inactive, increase tolerance, and escape hypoxia, and to prepare for arousal.

## 1. Introduction

Circadian rhythms are ubiquitous phenomena that drive daily biological rhythms of behavior and physiology, synchronizing them with periodic cycles of environmental cues [1,2]. Hibernation, as a common but important phenomenon in ectotherms [3], is characterized by inactivity and hypometabolism as a result of cold stress [4]. During normal conditions and hibernation periods, the circadian systems are involved in regulating endocrine and antioxidant defense systems in endothermic mammals [5,6]. Often, the daily rhythms of the clock genes stop in mammals during hibernation [5,6]. In ectotherms, recent findings show otherwise. Tissue-specific rhythms of clock genes and physiological parameters persist in some hibernating terrestrial ectotherms [7,8]. Although this is exciting, it remains enigmatic. Unlike mammals, how ectotherms exhibit physiological circadian rhythms and how their circadian clock genes regulate the endocrine and antioxidant defense systems in light-dark cycles under natural conditions and how they adjust this regulation during hibernation, remain largely unexplored [9].

The molecular circadian clock of tetrapods is highly conserved. It is characterized by an interlocked transcriptional/translational feedback loop comprising the core clock and their clock-controlled genes [2,10]. Brain and muscle arnt-like 1 (*Bmal1*) drives all circadian pacemakers by heterodimerizing with circadian locomotor output cycles kaput (*Clock*) to serve as the transcription factor to other members of the core clock loop, cryptochrome (*Cry*) and period (*Per*), and many other clock-controlled genes [10,11]. Nuclear retinoic acid-related orphan receptor-α (*Ror-α*), a key clock-controlled gene, in turn, regulates the expression of *Bmal1* and its heterodimeric partner [12,13]. In terrestrial hibernators, these clock and clock-controlled gene rhythms cease either centrally, peripherally, or both after the hibernators lose cyclic environmental signals such as light and/or temperature, for example, in ectotherms [7,8,14,15,16] or endotherms [5,6,17]. Still, some terrestrial hibernators under constant darkness can maintain tissue-specific molecular clocks [8]. Unlike terrestrial hibernators, aquatic hibernators stay underwater, with periodic cycles of light signals [18,19]. How the aquatic hibernators modulate their tissue-specific molecular clocks remains poorly understood.

The circadian and endocrine systems interact to orchestrate physiological responses to optimize fitness in response to distinct environmental conditions [20,21]. The daily cycles of circulating corticosterone (CORT) and melatonin (MT) result from interactions with the circadian system [22,23,24,25]. Daily rhythms of CORT help the suprachiasmatic nuclei (SCN) in resetting peripheral clocks even in constant environments, such as the absence of light and food [22,23]. CORT also mediates metabolic homeostasis by acting on *Per* genes expression levels [22,24]. In frogs, the rate-limiting enzyme of MT, arylalkylamine N-acetyltransferase (AANAT), mainly comes from the eyes [25,26]. MT suppresses the transcription of *Ror-α* by activating its membrane receptor, Mel-1, consequently reducing the nocturnal *Bmal1* and *Clock* expression [27,28,29]. Among the membrane receptors *Mel-_1a_*, *Mel-_1b_* and *Mel-_1c_*, *Mel-_1c_* is commonly found in amphibians [30,31]. Although CORT and MT are critical for maintaining circadian rhythmicity, they may not occur [32,33,34,35,36] or be rhythmic simultaneously, for example, plasma CORT of hibernating terrestrial Asiatic toads (*Bufo gargarizans*) remained rhythmic, whereas plasma MT did not [8].

Circadian and endocrine systems regulate the antioxidant defense systems that utilize antioxidant enzymes (AOEs) and non-enzymes (e.g., MT) to scavenge free radicals [37,38]. Because the AOEs, i.e., superoxide dismutases (SOD), catalase (CAT), and glutathione peroxidase (GPx) [39,40], possess Ror-α response elements in their promoter regions, Ror-α can regulate both their transcriptional and protein levels [28,41,42]. Under stress such as hypoxia, MT is thought to stimulate the transcription of antioxidants but also enhance their efficiency. This is possible because MT, apart from its chronobiotic properties, is a highly efficient antioxidant [43,44]. For example, in rats and tree frogs (*Hypsiboas faber*), during stress, elevated CORT stimulates MT synthesis, and then MT helps to optimize the antioxidative defense system to counter the stress. Hence, stressed ectotherms (fish and frogs) and endotherms (mice) may exhibit significant circadian fluctuations of AOEs in either the brain or the liver or both [44,45,46,47,48]. To date, limited information is available on the circadian rhythms of AOEs in ectotherms and endotherms during hibernation.

Aquatic hibernators have moist, warm, light conditions, but can experience more hypoxia and are less tolerable to freezing temperatures [49,50]. These experiences prompt these hibernators to move sporadically to better microenvironments denoting some level of alertness even while hibernating [51,52,53]. By contrast, terrestrial hibernators encounter less hypoxia on average, but are stationary, inhabit darker and colder environments, and lose clock gene rhythms centrally and mostly peripherally [7,8,54,55]. These differing selection pressures may shape how each respond to these challenges. Whether aquatic ectotherms retain their central and peripheral clock gene rhythms during normal and hibernating conditions and whether these clocks drive the antioxidative defense system by the MT pathway are fundamental for understanding the physiological basis of ectothermic hibernation. Nevertheless, these questions remain largely unclear.

Giant spiny frogs (*Quasipaa spinosa*, family Dicroglossidae) are nocturnal species that are mainly distributed in central and southern China [56,57,58]. They are typical stream-dwelling frogs that breed in streams from April to October and hibernate underwater from November to March; this makes them suitable for studying the interaction of circadian, endocrine, and antioxidative defense systems during normal conditions and hibernation. Here, from the retina, brain, and liver of the giant spiny frogs under normal conditions and during hibernation, we investigated the mRNA expression of the core clock genes (*Clock*, *Bmal1*, *Cry1* and *Per2*), clock-controlled genes (*Ror-α*, *Mel-1c* and *AANAT*), antioxidant enzyme genes (*SOD1*, *SOD2*, *CAT* and *GPx*), and the levels of plasma MT and CORT, over a 24 h period at six intervals. Considering that during hibernation, light signals can still be perceived and infrequent sporadic movements are still carried out in aquatic ectotherm hibernators, we hypothesize that, in spiny frogs, the retina and brain clock and clock-controlled genes, relative to the liver, would sustain active circadian rhythms to stimulate endocrine clocks and antioxidant enzyme expression.

## 2. Materials and Methods

### 2.1. Animals and Tissue Sampling

Ninety-six adult giant spiny frogs (2–3 years old, body weight of approximately 75 ± 10 g) were used. They were obtained from a commercial breeding farm in Jinhua, Southeast China, located within their natural geographical distribution range. The frogs were maintained outdoors to simulate their natural ecological conditions with minimal human intervention. Stream water was used on the farm. Shelters were provided by rocks, black sponges, and aquatic plants under a natural light photoperiod. Frogs were fed daily with live mealworms (*Tenebrio molitor*) *ad libitum* in the late afternoon during the active season.

Pre-hibernating samples were collected on 30 October 2014 (sunrise at 6:12 am and sunset at 17:25 pm, ambient air temperature of 17.9 °C ± 1.6 °C, and water temperature 16.0 °C ± 0.5 °C). Hibernating samples were collected on 14 January 2015 (sunrise at 6:56 am, sunset at 17:18 pm, ambient air temperature of 4.0 °C ± 0.5 °C, and water temperature 2.0 °C ± 1 °C). For each group, adult frogs were harvested every 4 h starting from zeitgeber time (ZT2) over a 24 h period (2, 6, 10, 14, 18, and 22; *n* = 8 at each time-point). Rapid euthanization by decapitation soon followed to minimize acute changes in gene expression and stress hormone secretion. Immediately, peripheral blood samples were collected in centrifuge tubes, and thereafter treated with heparin for anticoagulation. Surgically, tissues of the brain (mostly the hypothalamus and diencephalon), retina, and liver were sampled and put in RNAlater solution (Thermo Fisher Scientific, Waltham, MA, USA) for preservation. As light affects circadian rhythms considerably, all sampling procedures were carried out under dark conditions (dim red light, <1lx). Then, the tissue samples were preserved at −80 °C pending further assays. Approval for all protocols were sought from the Committee on Animal Care and Use and the Committee on Specialty Aquaculture Institute of both Zhejiang Academy of Agricultural Sciences and Hebei Normal University, China.

### 2.2. Molecular Cloning

Isolation of total RNA from the retina, brain, and liver was done using an RNApure Tissue and Cell Kit (Cowin Biosciences Inc., Taizhou, China), following the manufacturer’s protocol. Using ultraviolet spectrophotometry at 260 and 280 nm, extracted RNAs were quantified (NanoDrop, Thermo Fisher Scientific, Waltham, MA, USA). Evaluation of RNA integrity was conducted using the ratio of 260/280 nm and non-denaturing agarose gel electrophoresis (Bio-Rad Laboratories, Hercules, CA, USA). Reverse transcription of the total RNA into complementary DNA (cDNA) was carried out using a SuperRT cDNA Synthesis Kit (Cowin Biosciences Inc., Taizhou, China), following the manufacturer’s instruction. The Primer v.5 software (Premier Biosoft International, San Francisco, CA, USA) was employed to design degenerated primers based on highly conserved regions determined by the ClustalW algorithm from the available vertebrate sequences for: four (4) core circadian clock genes (*Clock*, *Bmal1*, *Cry1* and *Per2*), clock-controlled genes (*Ror-α*, *Mel-1c* and *AANAT*), and AOE genes (*SOD1*, *SOD2*, *CAT and GPx*) (Table S1). Products of purified polymerase chain reaction (PCR) were ligated into a pUC18-T vector (Cowin Biosciences Inc., Taizhou, China). Then, the ligated products were transformed into *Escherichia coli* DH5α cells. After obtaining positive clones, plasmid DNA was extracted using a QIAprep Spin Miniprep Kit (Qiagen, Hilden, Germany). Using Sanger sequencing, nucleotide sequences were determined, and then confirmed by bioinformatics analysis.

### 2.3. Quantitative Real-Time PCR

Quantitative real-time PCR (qPCR) was performed using a SYBR Premix Ex Taq II Kit (Clontech Laboratories, Palo Alto, CA, USA) on a StepOnePlus Real-Time PCR System (Applied Biosystems, Foster City, CA, USA). The primers for each of the target genes (Table S1) were designed by employing the Primer Express Software Version 3.0 (Thermo Fisher Scientific, Waltham, MA, USA) based on the partial sequence of each target gene and synthesized by Invitrogen Corporation (Shanghai, China). The specificity of the primer pairs for each target gene was verified using a melting curve analysis. PCR was performed in a 200 µL microfuge tube containing 0.4 µL of each primer (10 μmol/L), 10 µL SYBR Premix Ex Taq II (contains Taq DNA polymerase, reaction buffer, dNTP mix, and 10 mM MgCl_2_), 0.2 µL ROX Reference Dye (50×), 7 µL ddH_2_O, and 2 µL cDNA in a total volume of 20 µL. The amplification reaction conditions were set as follows: initial denaturation and enzyme activation at 95 °C for 1 min, 45 cycles of denaturation at 95 °C for 15 s, and annealing and extension at 60 °C for 30 s. To determine the relative mRNA expression of all genes analyzed, the transcript levels of each gene in each sample were calculated using the 2 ^ΔΔ^Ct methods. Endogenous relative expression was normalized to β-actin (internal control) and the fold-change from the control group CT value [59].

### 2.4. Measurements of Plasma MT and CORT Concentrations

Determinations of plasma MT and CORT levels were done using enzyme-linked immunosorbent assay kits for amphibians (Nanjing Jiancheng Bioengineering Institute, Nanjing, China) having a sensitivity of 0.05 ng/mL and intra- and inter-assay variation coefficients of 9% and 11%, respectively. Then, 50 μL of plasma was diluted 5-fold before the assays. MT and CORT in the samples and standards competed with biotinylated MT and CORT for binding to solid-phase antibody-coated 96-well assay plates. Streptavidin-conjugated enzyme was added for detection, and the substrate chromophore was formed in inverse proportion to the amount of plasma MT and CORT in the sample/standard. The intensity of the immunosorbent of each well in the assay plate was determined using spectrophotometry at 450 nm using a standard plate reader (M200 pro, Tecan, Switzerland). Samples were all run in duplicates.

### 2.5. Statistical Analyses

A generalized model was used to determine the effects of treatment, time, tissue, and their interactions on *Clock*, *Bmal1*, *Cry1*, *Per2*, *Ror-α*, *Mel_1c_*, *SOD1*, *SOD2*, *CAT* and *GPx* genes; and the effects of treatment and time and their interactions on plasma MT, CORT, and retinal *AANAT*. Furthermore, the JTK_cycle package from R software [60] was employed to determine circadian rhythms and rhythm parameters (Mesor, Amplitude, and Acrophase) of the genes, MT and CORT. Time-series curves of variables with significant circadian rhythms were drawn using fitted cosine waves. Bonferroni *post hoc* test followed multiple comparisons in each of the two group. Adjusted *p*-values (ADJ.P) < 0.05 indicate significant circadian rhythms. IBM SPSS Statistics 25 (IBM Inc., New York, NY, USA) was used for all statistical analyses, while all plots were done using GraphPad Prism 8.0 (GraphPad Software Inc., CA, USA). The results are expressed as the mean ± standard error.

## 3. Results

### 3.1. Rhythmicity of the Clock Genes

**Figure 1:**
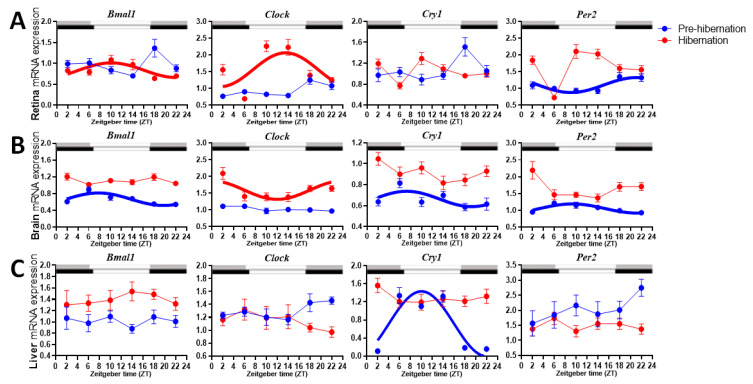
Comparison of mRNA expression levels of *Bmal1*, *Clock*, *Cry1* and *Per2* in: (**A**) retina; (**B**) brain; (**C**) liver, in the spiny frog (*Quasipaa spinosa*) pre-hibernation and during hibernation. Rhythms are deemed significant when the adjusted *p*-value (ADJ.P) is <0.05, and are presented in bold. The times of sunrise and sunset on the sampling days, which were used as daytime (open bar) and nighttime (gray bar for pre-hibernation and black bar for hibernation), were available from the local meteorological observatory.

Before commencing hibernation, whereas retinal *Bmal1*, *Clock* and *Cry1* expression were non-rhythmic, retinal *Per2* expression displayed rhythm that peaked at ZT20 (Table 1 and Figure 1). Brain *Bmal1*, *Per2* and *Cry1* were rhythmic, and their acrophases synchronized between ZT6 and ZT8, but *Clock* expression was not rhythmic. In the liver, only *Cry1* expression exhibited rhythm that had acrophase at ZT10. After entering hibernation, retinal *Bmal1* and *Clock* expression became rhythmic with acrophases at ZT8 and ZT14, respectively, whereas *Cry1* and *Per2* did not display any significant rhythms (Table 1 and Figure 1). In the brain, *Clock* expression became rhythmic with acrophase at ZT0, but *Bmal1*, *Cry1* and *Per2* expressions became unrhythmic. In the liver, *Cry1* expression became unrhythmic, and *Clock*,* Bmal1* and *Per2* expressions remained unrhythmic. By comparing clock genes expression before hibernation and during hibernation, it revealed that retina *Clock* and *Per2*; brain *Clock*, *Bmal1*, *Cry1* and *Per2*; and liver *Bmal1* with *Cry1* were significantly upregulated, and that liver *Clock* and *Per2* were significantly downregulated in hibernating frogs (Table 2; Figure 1).

### 3.2. Rhythmicity of Ror-α, AANAT, and Mel-1c Genes

**Figure 2:**
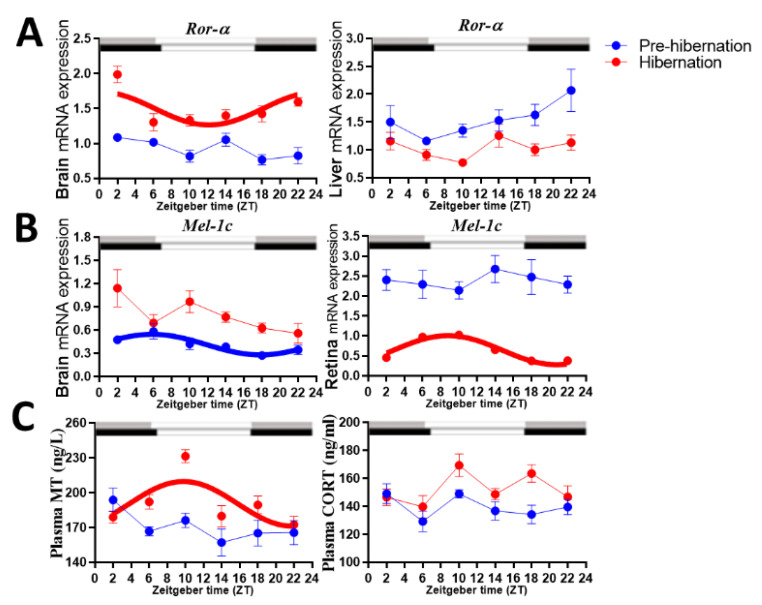
Comparison of mRNA expression levels of: (**A**) *Ror-α* in the brain and liver; (**B**) *Mel-1c* in the retina and brain; (**C**) comparison of plasma melatonin (MT) and corticosterone (CORT) in the spiny frog (*Quasipaa spinosa*) pre-hibernation and during hibernation. Rhythms are deemed significant when the adjusted *p*-value (ADJ.P) is <0.05, and are presented in bold. The times of sunrise and sunset on the sampling days, which were used as daytime (open bar) and nighttime (gray bar for pre-hibernation and black bar for hibernation), were available from the local meteorological observatory.

Prior to hibernation, brain, and liver *Ror-α* expression levels were non-rhythmic (Table 1 and Figure 2). Retinal *AANAT* and *Mel-1c* expression were not rhythmic, however, brain *Mel-1c* was rhythmic with a peak at ZT6. While the frogs were hibernating, brain *Ror-α* expression became rhythmic with an acrophase at ZT0, and liver *Ror-α* remained non-rhythmic (Table 1 and Figure 2). Retina *AANT* expression remained non-rhythmic, but retinal *Mel-1c* expression became rhythmic with an acrophase at ZT8. By comparing the clock-related genes expression levels before entering hibernation and during hibernation groups, it revealed significant *Ror-α* upregulation in the brain and downregulation in the liver (Table 2 and Figure 2A). Likewise, significant upregulation of *Mel-1c* expression was seen in the brain and downregulation in the retina (Figure 2B). Surprisingly, retinal *AANAT* expression levels were similar in both conditions (Figure S1).

### 3.3. Rhythmicity of Plasma MT and CORT Levels

MT and CORT levels were non-rhythmic during the pre-hibernation period. However, MT became rhythmic with an acrophase at ZT8, and CORT remained unrhythmic during the hibernation period (Table 1 and Figure 2C). MT and CORT levels both increased significantly during the hibernation period as compared with the pre-hibernation period (MT, *t* = −3.208 and *p* = 0.002; CORT, *t* = −3.006 and *p* = 0.004).

### 3.4. Rhythmicity of the AOEs

**Figure 3:**
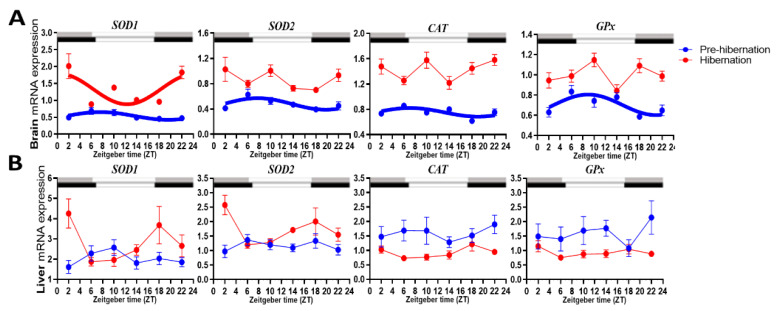
Comparison of mRNA expression levels of the antioxidant enzymes *SOD1*, *SOD2*, *CAT* and G*Px* in: (**A**) brain; (**B**) liver, of spiny frog (*Quasipaa spinosa*) pre-hibernation and during hibernation. Rhythms are deemed significant when the adjusted *p*-value (ADJ.P) is <0.05, and are presented in bold. The times of sunrise and sunset on the sampling days, which were used as daytime (open bar) and nighttime (gray bar for pre-hibernation and black bar for hibernation), were available from the local meteorological observatory.

Under the normal condition, *SOD1*, *SOD2*, *CAT* and *GPx* gene expression was rhythmic in the brain, with synchronized acrophases between ZT6 and ZT8, but they were unrhythmic in the liver (Table 1 and Figure 3). During the hibernation period, only brain *SOD1* remained rhythmic with an acrophase at ZT0 and *SOD2*, *CAT* and *GPx* became unrhythmic, while the four genes remained unrhythmic in the liver. None of the antioxidant enzyme transcripts were detected in the retina. By comparing antioxidant enzyme gene expression levels, it showed that all the AOEs were significantly upregulated in the brains of the hibernating frogs (Table 2 and Figure 3). In the liver, the hibernating frogs significantly upregulated *SOD1* and *SOD2*, while *CAT* and *GPx* were downregulated.

## 4. Discussion

The high synchronizations in the retina and the brain that we observed from this study support our hypothesis that retina and brain clocks, together with the clock-controlled genes, help to sustain the endocrine clock and the antioxidant enzyme expression in an aquatic hibernator. In the hibernating frogs, plasma MT unlikely drove AOEs, which differs from the findings in mammals [28,38]. Meanwhile, circadian rhythms of retina clocks and *Mel-1c*, and brain *Ror-α*, but not plasma CORT may contribute to maintaining their characteristics of inactivity and hypometabolism underwater, which totally differs from those central and peripheral circadian clocks in terrestrial hibernators.

### 4.1. Rhythmicity of Clock Genes, Clock-Controlled Genes, CORT, and MT

Under normal conditions, retina *Per2* reached acrophase (its peak) during the night. Brain *Bmal1* stimulated the observed rhythms of the genes that culminated in their synchronizations within 2 h of sunrise (daybreak), and liver *Cry1* 2 h later. Similarly, non-hibernating tree frogs (*Polypedates teraiensis*) had robust brain *Bmal1* and *Per2* rhythms [7], and pre-hibernating European hamsters (*Cricetus cricetus*) had rhythmic brain *Per2* expression. In mammals, *Cry1* provides temporal downregulation of gluconeogenesis [61]; thus, the rhythmicity we observed of brain and liver *Cry1* and brain *Bmal1* suggests a significant contribution to an oscillation of energy metabolism, when the frogs are in their resting periods. Retinal *AANAT*, and plasma MT levels were non-rhythmic, even though brain *Mel-1c* was rhythmic with acrophases at night. Our results are consistent with those observed in the European green toad (*Bufo viridis*), which presented non-rhythmic retinal *AANAT* [62]. Considering that the giant spiny frogs are nocturnal animals, *Per2* may enhance carbohydrate and lipid metabolism (to aid foraging and navigation) [63], and *Mel-1c* may boost MT activities to maintain synchronized core and peripheral clocks [27,28,29]. The stimulated genes at daybreak may also boost activities that could reduce oxidative stress associated with a high metabolism from the previous night [64,65,66].

While before hibernation there was synchronizations primarily in the brain, during hibernation, there were synchronizations in the retina driven by *Bmal1* after daybreak. There were synchronizations in the brain most likely driven by *Clock* in the night. This is consistent with hibernating terai tree frogs (a terrestrial species) that had robust circadian fluctuations of the brain *Clock* while losing liver clocks [7]. Our results, however, differ from the findings of complete loss of all clock gene rhythms in the brain and the retina of terrestrial hibernating ectotherms (e.g., Asiatic toads [8]) and brain of endotherms (e.g., European hamsters [5,6] and Arctic ground squirrels *Spermophilus parryi* [67,68,69]). Our results showed that brain *Ror-α* and *Clock* in hibernating frogs were phase advanced by approximately 8 h relative to pre-hibernation, with upregulated brain core clock and clock-controlled genes. This indicates that aquatic frogs may retain some of their endogenous circadian rhythms during a long period of hibernation, although they are decoupled mainly by their external environments of cold climate. Given that Ror-α induces the hypoxia signaling pathway [41], the involvement of hypoxia-inducible factor-α in regulating the *Ror-α* transcriptional profile to increase hypoxia tolerance in hibernating aquatic frogs warrants further investigation.

Still, during hibernation, retina *Bmal1* and *Mel-1c* and plasma MT levels in the frogs were highly rhythmic and synchronized at ZT8 (daytime), while none of the negative limb clock genes was rhythmic. Such remarkable and synchronized rhythmicity in the retina and shift to light periods of plasma MT during hibernation suggests that the retina remained active. These results are novel and unexpected because, at cold temperatures, plasma MT is commonly upregulated with arrested oscillations, and primarily functions to mitigate the negative effects of injuries associated with arousal from hibernation. Such phenomenon has been reported in hibernating frogs (*Rana perezi*), European hamsters, and marmots (*Marmota flaviventris*) [5,70,71]. Furthermore, brain *Mel-1c* of hibernating frogs was non-rhythmic but upregulated, whereas retinal *Mel-1c* was rhythmic but downregulated. These results confirm that the MT receptor is a clock-controlled element. By perceiving external light dark cycles in the retina with a relatively conscious brain (*Clock* and *Ror-α*) to enable spontaneous and non-periodic movements in the water environment, the rhythmicity of MT levels may have benefited the brain and retina for antioxidative purposes.

Surprisingly, high synchronization was observed between brain *Bmal1* and *Mel-1c* and plasma MT, but not plasma CORT, in the hibernating giant spiny frogs. This is unexpected, and is in contrast to the remarkable rhythmicity of CORT in pre-hibernation and during hibernation but not MT in terrestrial Asiatic toads [8] and lost MT rhythms in the marmots [70]. As circulating MT mainly comes from adult frog eyes [25], our results indicate that the retina significantly contributed to MT rhythms because retinal *Clock*/*Bmal1* activates *AANAT* [72], despite the retinal *AANAT* being non-rhythmic. In non-mammals, the retina functions substantially as a multi-oscillator pacemaker; therefore, terrestrial hibernating ectotherms may lose MT rhythms because the retina does not encounter cyclic light signals, unlike aquatic ones that can still perceive light. Notably, the giant spiny frogs showed increased CORT and MT levels during hibernation. Intact adrenal clock gene rhythms are necessary for CORT level rhythms [24,73] under normal conditions and hibernating periods, which induce MT secretion [45]. Whether increased CORT levels contribute to maintaining liver metabolic capacity and increased MT levels contribute to preventing oxidative stress remain to be investigated.

### 4.2. Rhythmicity of Antioxidant Enzyme Genes

During pre-hibernation, the significant rhythms displayed by brain *SOD1*, *SOD2*, *CAT* and *GPx* were highly synchronized with the rhythms of brain *Bmal1*, *Cry1* and *Per2*, and liver *Cry1* between ZT6 and ZT10. Our results are in line with the notion that rhythmicity of *Per2* is essential for *SOD1* rhythm in mammals [39,74]. Similarly, mice exhibited significant brain *CAT* circadian rhythms but not liver *CAT* activity [40]. Furthermore, the peaks we observed of AOEs in the brain and liver were mostly during the dark periods, corresponding to nocturnality of giant spiny frogs. During hibernation, only brain *SOD1* was significantly rhythmic and highly synchronized to brain *Ror-α* with acrophases at ZT0 (night) and almost 120 °C out-of-phase with MT rhythm with an acrophase at ZT8 (day). Furthermore, increased *Bmal1* expression in the hibernating group correlated with increased AOEs in the brain and liver, suggesting that *Bmal1* increased *Ror-α* expression with increasing ROS. We did not detect AOEs or significant *Bmal1* transcripts in the retina, suggesting that frogs may not suffer from retinal oxidative stress [74]. This is unlike mammals, where retinal Bmal1 deficiency results in higher ROS [75]. However, hibernating frogs had rhythmic retinal *Bmal1* and *Clock*, with high expression of *Clock*. Considering that retinal function should be reduced significantly under suppressed activity during hibernation, we speculate that *Bmal1*:*Clock* might effectively suppress the generation of ROS [74].

Many amphibians that overwinter underwater tolerate prolonged bouts of hypoxia, and therefore, are prone to oxidative stress [47,48]. Hence, overall, their antioxidant metabolites increase [76], but in a tissue-specific fashion. Our results show that the hibernating spiny frogs upregulated both the first line of defense (*SOD*) and second line of defense (*CAT* and *GPx*) in the brain; they upregulated first line of defense and downregulated second line of defense in the liver. Interestingly, in a similar aquatic hibernating frog from a high altitude, the Tibetan plateau frog (*Nanorana parkeri*), the results showed that these frogs downregulated first line of defense in the liver, but they also downregulated second line of defense in the brain [77,78]. The authors reported low hepatic ROS, despite significant hypoxia in this species environment and hibernacula [79]. In the terrestrial hibernating ground squirrels, first line of defense activity increased in both the brain and the liver [80], similar to the giant spiny frogs. The selective upregulation and/or downregulation of antioxidant defense activities in the brain and liver suggest that the extent of oxidative stress may determine these changes and has less to do with the animals’ hibernacula. Still, all successful hibernators reach common goal- maintaining tolerable ROS. For example, our findings show that the spiny frogs upregulated first line of defense and downregulated second line of defense in the liver-suggesting superoxide radicals were minimized. The upregulated AOEs, especially in the brain, may be a pointer to hypoxia tolerance, as cell proliferation counters brain cell apoptosis probably mediated by calcium binding proteins, as adaptive and neuroprotective strategies [81]. All these strategies would protect against immediate stress and injury resulting from arousal, as observed in lake frogs (*Rana ridibunda*) [82].

Daily fluctuations of physiological variables, such as AOEs, may be under the combined control of exogenous and endogenous factors, for esample, decoupling the variables from the control of circadian system [40]. Our inability to detect significant rhythms of AOEs in the retina and liver does not completely exclude the possibility that the circadian system modulates these rhythms. More importantly, most studies in vertebrates reporting circadian variations in AOEs do not qualify as circadian rhythms; the rhythms are rather ultradian, for esample, in the black sea bass (*Centropristis striata*), African clawed frog (*Xenopus laevis*), mice, rats, and ground squirrels [40,47,83,84]. Pre-hibernation and during hibernation, AOE transcripts had irregular ultradian spikes spanning day and night. This might be a result of the demands of the moments driving the expression (upregulation and downregulation) of AOE transcripts rather than a temporal coincidence [40]. Some genes may only exhibit significant rhythms after post-translational modifications [85]; therefore, further research is needed to determine whether AOE protein levels are rhythmic in hibernating ectotherms.

## 5. Conclusions

We found that brain *Bmal1* of the giant spiny frogs drove the expression of many other clock genes under normal conditions, and these genes synchronized and reached their peaks around 2 h after daybreak. During hibernation, two separate synchronizations occurred: one in the retina driven by *Bmal1* immediately after daybreak, the second one in the brain driven by *Clock* in the night. Our results point to strong circadian rhythm signals that exist in the central and peripheral organs of the neurosensory and metabolic systems in nocturnal aquatic frogs. These signals are adjusted principally by the cyclic light inputs, and to a lesser extent by the cold temperature by phase-advancing the molecular clock. Unlike mammals in which cold temperatures abolish their molecular clocks, this study and our previous findings on Asiatic toads point to two possible reasons for this tissue-specific phenomena. First, cold temperatures decouple, and not abolish, the multi-oscillator pacemakers of non-mammals, enabling the oscillators to function independently. Second, it seems amphibian hibernators remain largely unconscious during their prolonged hibernation that differs from exhibiting interbout arousals to full euthermic states in mammals. Therefore, tissue-specific rhythmic molecular clock may temporally regulate critical basic physiological processes that ensure survival and arousal.

Under normal conditions, we found lack of rhythms of plasma CORT and in the liver clocks, except liver *Cry1* and brain *Mel-1c*, indicating that the rhythms of antioxidant enzymes are not driven by metabolic organs or hormones, which tend to be driven by MT receptors in the neurosensory system. During hibernation, our results suggest that the retina *Bmal1*, *Clock*, *Mel-1c* and brain *Clock* of aquatic frogs remained rhythmically active, but not those in the liver, which is highly associated with their hibernating underwater environments with cyclic lights in contrast to terrestrial hibernating toads under constant darkness in the burrows. Notably, these frogs’ brain *Ror-α* and highly synchronized rhythms and fluctuations were similar to that of the antioxidant enzymes (*SOD1*) and almost opposite that of plasma MT. This indicates that plasma MT does not seem to drive the AOEs even under hibernation, as obtained in mammals. The adaptive strategies employed by amphibian hibernators are quite remarkable. Aquatic hibernators such as the giant spiny frogs need a retina clock for navigation and a brain clock for coordinating their compulsive movements. This strategy differs from terrestrial hibernators such as the Asiatic toads, as they only need rhythmic hepatic and adrenal clocks to ensure metabolic oscillators until they reach arousal when they are stationary under borrows. Our findings improve our understanding regarding the ecophysiological strategy of how aquatic ectotherms cope with cold and water environments during hibernating and non-hibernating stages by linking clock circadian systems, plasma hormones, and antioxidative systems.

## Figures and Tables

**Table 1:** Circadian rhythm parameters of mRNA expression of *Bmal1*, *Clock*, *Cry1* and *Per2* in brain, retina, and liver; *SOD1*, *SOD2*, *CAT*, *GPx* and *Ror-α* in the brain and the liver; *Mel_1c_* in the brain and retina; *AANAT* in retina; and plasma MT and CORT of the giant spiny frog (*Quasipaa spinosa*) pre-hibernation and during hibernation.

Pre-Hibernation					Hibernation				
Tissue	Variable	Mesor	Amplitude	Acrophase	ADJ.P	Mesor	Amplitude	Acrophase	ADJ.P
Retina	*Bmal1*	0.97	0.03	0	0.659	**0.84**	**0.13**	**8**	**0.004**
	*Clock*	0.94	0.11	20	0.144	**1.59**	**0.56**	**14**	**0.009**
	*Cry1*	1.07	0.11	18	0.194	1.05	0.09	12	1.000
	*Per2*	**1.10**	**0.21**	**20**	**0.011**	1.65	0.36	14	0.135
Brain	*Bmal1*	**0.67**	**0.13**	**8**	**<0.001**	1.12	0.04	12	1.000
	*Clock*	1.03	0.04	2	0.360	**1.59**	**0.13**	**0**	**0.027**
	*Cry1*	**0.66**	**0.04**	**6**	**0.031**	0.92	0.06	2	0.102
	*Per2*	**1.05**	**0.13**	**8**	**0.002**	1.65	0.18	0	0.057
Liver	*Bmal1*	1.02	0.07	22	1.000	1.59	0.13	12	0.816
	*Clock*	1.30	0.09	22	0.384	1.15	0.09	10	1.000
	*Cry1*	**0.68**	**0.72**	**10**	**<0.001**	1.29	0.10	0	0.851
	*Per2*	2.63	0.32	18	1.000	1.48	0.09	10	1.000
Brain	*SOD1*	**0.54**	**0.06**	**6**	**0.033**	**1.33**	**0.21**	**0**	**0.022**
	*SOD2*	**0.48**	**0.07**	**8**	**0.035**	0.87	0.09	4	0.570
	*CAT*	**0.75**	**0.04**	**6**	**0.022**	1.43	0.03	0	0.881
	*GPx*	**0.70**	**0.13**	**8**	**0.014**	1.00	0.04	6	1.000
Liver	*SOD1*	2.03	0.25	10	0.748	2.82	0.64	22	0.450
	*SOD2*	1.17	0.10	10	1.000	1.72	0.33	20	0.558
	*CAT*	1.59	0.17	0	1.000	0.92	0.10	18	0.160
	*GPx*	1.60	0.10	16	1.000	0.94	0.13	22	1.000
Brain	*Ror-α*	0.93	0.08	4	0.270	**1.50**	**0.16**	**0**	**0.007**
Liver	*Ror-α*	1.54	0.22	18	0.194	1.04	0.13	20	0.310
Retina	*Mel-1c*	2.38	0.12	18	1.000	0.64	0.36	8	**<0.001**
Brain	*Mel-1c*	**0.41**	**0.10**	**6**	**0.007**	0.79	0.07	8	0.191
Retina	*AANAT*	1.39	0.19	18	0.816	1.05	0.23	10	0.659
Plasma	MT	170.93	4.76	2	0.305	**190.41**	**14.22**	**8**	**0.006**
	CORT	139.54	4.55	6	1.000	151.95	2.08	16	0.620

Rhythms are deemed significant if the adjusted *p*-value (ADJ.P) is <0.05, and are presented in bold.

**Table 2:** Comparison between mRNA expression levels of core clock genes (*Bmal1*, *Clock*, *Cry1* and *Per2*), clock-controlled genes (*Ror-α*, *Mel-1c* and *AANAT*), and antioxidant enzymes (*SOD1*, *SOD2*, *CAT* and *GPx*) pre-hibernation and during hibernation using independent *t*-tests in giant spiny frogs (*Quasipaa spinosa)*.

Parameters	Variables	Retina		Brain		Liver	
		*T* Value	*p* Value	*t* Value	*p* Value	*t* Value	*p* Value
*Clock genes*	*Bmal1*	1.947	0.055	−11.963	**<0.001**	−5.761	**<0.001**
	*Clock*	−5.672	**<0.001**	−8.598	**<0.001**	2.139	**0.035**
	*Cry1*	0.118	0.906	−7.739	**<0.001**	−5.328	**<0.001**
	*Per2*	−5.423	**<0.001**	−7.741	**<0.001**	4.896	**<0.001**
*Clock controlled genes*	*Ror-α*			−8.987	** <0.001 **	4.325	**<0.001**
	*Mel-1c*	13.294	**<0.001**	−5.63	**<0.001**		
	*AANAT*	1.832	0.07				
*Antioxidants*	*SOD1*			−7.994	**<0.001**	−2.784	**0.007**
	*SOD2*			−7.779	**<0.001**	−3.805	**<0.001**
	*CAT*			−13.957	**<0.001**	4.573	**<0.001**
	*GPx*			−7.99	**<0.001**	3.657	**0.001**

Values that are statistically significant are presented in bold.

## Data Availability

We will upload original data to the public database of Figshare.

## Supplementary Materials

The following supporting information can be downloaded at https://www.mdpi.com/article/10.3390/biology11050722/s1, Table S1: Primer sequences used for cloning genes and for qPCR analysis, Figure S1: Comparison of mRNA expression of retinal *AANAT* in the giant spiny frog (*Quasipaa spinosa*) between pre-hibernation and during hibernation. Rhythms are deemed significant when the adjusted *p*-value (ADJ.P) is <0.05. The times of sunrise and sunset on the sampling days, which were used to graph the daytime (open bar) and nighttime (gray bar for pre-hibernation and black bar for hibernation), were available from the local meteorological observatory.

## References

[1] Kumar V. (2002). Biological Rhythms.

[2] Takahashi J.S. (2017). Transcriptional architecture of the mammalian circadian clock. Nat. Rev. Genet..

[3] Miller N. (1909). The American toad (Bufo Lentiginosus Americanus, LeConte). II A Study in dynamic biology. Am. Nat..

[4] Ultsch G.R., Reese S.A., Stewart E.R. (2004). Physiology of hibernation in *Rana pipiens*: Metabolic rate, critical oxygen tension, and the effects of hypoxia on several plasma variables. J. Exp. Zool..

[5] Revel F.G., Herwig A., Garidou M.-L., Dardente H., Menet J.S., Masson-Pévet M., Simonneaux V., Saboureau M., Pévet P. (2007). The circadian clock stops ticking during deep hibernation in the European hamster. Proc. Natl. Acad. Sci. USA.

[6] Gautier C., Bothorel B., Ciocca D., Valour D., Gaudeau A., Dupré C., Lizzo G., Brasseur C., Riest-Fery I., Stephan J.-P. (2018). Gene expression profiling during hibernation in the European hamster. Sci. Rep..

[7] Borah B.K., Renthlei Z., Trivedi A.K. (2020). Hypothalamus but not liver retains daily expression of clock genes during hibernation in terai tree frog (*Polypedates teraiensis*). Chronobiol. Int..

[8] Xie Z., Ahmad I.M., Zuo L., Xiao F., Wang Y., Li D. (2021). Hibernation with rhythmicity: The circadian clock and hormonal adaptations of the hibernating Asiatic toads (*Bufo gargarizans*). Integr. Zool..

[9] Withers P., Cooper C.E. (2019). Dormancy. Encycl. Ecol..

[10] Cox K.H., Takahashi J.S. (2019). Circadian clock genes and the transcriptional architecture of the clock mechanism. J. Mol. Endocrinol..

[11] Mohawk J.A., Green C.B., Takahashi J.S. (2012). Central and peripheral circadian clocks in mammals. Annu. Rev. Neurosci..

[12] Akashi M., Takumi T. (2005). The orphan nuclear receptor RORα regulates circadian transcription of the mammalian core-clock Bmal1. Nat. Struct. Mol. Biol..

[13] Mazzoccoli G., Pazienza V., Vinciguerra M. (2012). Clock Genes and Clock-Controlled Genes in the Regulation of Metabolic Rhythms. Chronobiol. Int..

[14] Gatten R.E. (1987). Cardiovascular and Other Physiological Correlates of Hibernation in Aquatic and Terrestrial Turtles. Am. Zool..

[15] Magnone M.C., Jacobmeier B., Bertolucci C., Foà A., Albrecht U. (2005). Circadian expression of the clock gene Per2 is altered in the ruin lizard (*Podarcis sicula*) when temperature changes. Mol. Brain Res..

[16] Vallone D., Frigato E., Vernesi C., Foà A., Foulkes N.S., Bertolucci C. (2007). Hypothermia modulates circadian clock gene expression in lizard peripheral tissues. Am. J. Physiol. Integr. Comp. Physiol..

[17] Ikeno T., Williams C., Buck C.L., Barnes B.M., Yan L. (2017). Clock Gene Expression in the Suprachiasmatic Nucleus of Hibernating Arctic Ground Squirrels. J. Biol. Rhythm..

[18] Yearicks E.F., Wood R.C., Johnson W.S. (1981). Hibernation of the Northern Diamondback Terrapin, *Malaclemys terrapin terrapin*. Estuaries.

[19] Ultsch G.R. (1989). Ecology and physiology of hibernation and overwintering among freshwater fishes, turtles, and snakes. Biol. Rev..

[20] Ikegami K., Refetoff S., Van Cauter E., Yoshimura T. (2019). Interconnection between circadian clocks and thyroid function. Nat. Rev. Endocrinol..

[21] Neumann A.-M., Schmidt C.X., Brockmann R.M., Oster H. (2019). Circadian regulation of endocrine systems. Auton. Neurosci..

[22] Oster H. (2020). The interplay between stress, circadian clocks, and energy metabolism. J. Endocrinol..

[23] Gamble K.L., Berry R., Frank S.J., Young M.E. (2014). Circadian clock control of endocrine factors. Nat. Rev. Endocrinol..

[24] So A.Y.-L., Bernal T.U., Pillsbury M.L., Yamamoto K.R., Feldman B.J. (2009). Glucocorticoid regulation of the circadian clock modulates glucose homeostasis. Proc. Natl. Acad. Sci. USA.

[25] Wright M.L. (2002). Melatonin, diel rhythms, and metamorphosis in anuran amphibians. Gen. Comp. Endocrinol..

[26] Isorna E., Besseau L., Boeuf G., Desdevises Y., Vuilleumier R., Alonso-Gómez A.L., Delgado M.J., Falcón J. (2006). Retinal, pineal and diencephalic expression of frog arylalkylamine N-acetyltransferase-1. Mol. Cell. Endocrinol..

[27] Venegas C., García J.A., Doerrier C., Volt H., Escames G., López L.C., Reiter R.J., Acuña-Castroviejo D. (2013). Analysis of the daily changes of melatonin receptors in the rat liver. J. Pineal Res..

[28] Hardeland R. (2018). Melatonin and retinoid orphan receptors: Demand for new interpretations after their exclusion as nuclear melatonin receptors. Melatonin Res..

[29] Li D.Y., Smith D.G., Hardeland R., Yang M.Y., Xu H.L., Zhang L., Yin H.D., Zhu Q. (2013). Melatonin Receptor Genes in Vertebrates. Int. J. Mol. Sci..

[30] Wiechmann A.F., Wirsig-Wiechmann C.R. (2001). Melatonin Receptor mRNA and Protein Expression in *Xenopus laevis* Nonpigmented Ciliary Epithelial Cells. Exp. Eye Res..

[31] Wiechmann A.F., Smith A.R. (2001). Melatonin receptor RNA is expressed in photoreceptors and displays a diurnal rhythm in Xenopus retina. Mol. Brain Res..

[32] Owino S., Buonfiglio D.D.C., Tchio C., Tosini G. (2019). Melatonin Signaling a Key Regulator of Glucose Homeostasis and Energy Metabolism. Front. Endocrinol..

[33] Hernández-Pérez J., Míguez J.M., Librán-Pérez M., Otero-Rodiño C., Naderi F., Soengas J.L., López-Patiño M.A. (2015). Daily rhythms in activity and mRNA abundance of enzymes involved in glucose and lipid metabolism in liver of rainbow trout, Oncorhynchus mykiss. Influence of light and food availability. Chronobiol. Int..

[34] Cahill G.M. (1996). Circadian regulation of melatonin production in cultured zebrafish pineal and retina. Brain Res..

[35] Falso P.G., Noble C.A., Diaz J.M., Hayes T.B. (2015). The effect of long-term corticosterone treatment on blood cell differentials and function in laboratory and wild-caught amphibian models. Gen. Comp. Endocrinol..

[36] Cahill G.M., Besharse J.C. (1993). Circadian clock functions localized in xenopus retinal photoreceptors. Neuron.

[37] Hill S.M., Frasch T., Xiang S., Yuan L., Duplessis T., Mao L. (2009). Molecular Mechanisms of Melatonin Anticancer Effects. Integr. Cancer Ther..

[38] Tomás-Zapico C., Coto-Montes A. (2005). A proposed mechanism to explain the stimulatory effect of melatonin on antioxidative enzymes. J. Pineal Res..

[39] Jang Y.-S., Lee M.-H., Lee S.-H., Bae K. (2011). Cu/Zn superoxide dismutase is differentially regulated in period gene-mutant mice. Biochem. Biophys. Res. Commun..

[40] Sani M., Sebaï H., Gadacha W., Boughattas N.A., Reinberg A., Mossadok B.A. (2006). Catalase activity and rhythmic patterns in mouse brain, kidney and liver. Comp. Biochem. Physiol. Part B Biochem. Mol. Biol..

[41] Kim E.-J., Yoo Y.-G., Yang W.-K., Lim Y.-S., Na T.-Y., Lee I.-K., Lee M.-O. (2008). Transcriptional Activation of HIF-1 by RORα and its Role in Hypoxia Signaling. Arterioscler. Thromb. Vasc. Biol..

[42] Zhao Y., Xu L., Ding S., Lin N., Ji Q., Gao L., Su Y., He B., Pu J. (2017). Novel protective role of the circadian nuclear receptor retinoic acid-related orphan receptor-α in diabetic cardiomyopathy. J. Pineal Res..

[43] Reiter R.J., Tan D.-X., Mayo J.C., Sainz R.M., Leon J., Czarnocki Z. (2003). Melatonin as an antioxidant: Biochemical mechanisms and pathophysiological implications in humans. Acta Biochim. Pol..

[44] Tan D.-X., Manchester L.C., Esteban-Zubero E., Zhou Z., Reiter R.J. (2015). Melatonin as a Potent and Inducible Endogenous Antioxidant: Synthesis and Metabolism. Molecules.

[45] Ward C.K., Fontes C., Breuner C.W., Mendonça M.T. (2007). Characterization and quantification of corticosteroid-binding globulin in a southern toad, *Bufo terrestris*, exposed to coal-combustion-waste. Gen. Comp. Endocrinol..

[46] Joanisse D.R., Storey K. (1996). Oxidative damage and antioxidants in *Rana sylvatica*, the freeze-tolerant wood frog. Am. J. Physiol. Integr. Comp. Physiol..

[47] Ren X., Zhang J., Wang L., Wang Z., Wang Y. (2020). Diel variation in cortisol, glucose, lactic acid and antioxidant system of black sea bass *Centropristis striata* under natural photoperiod. Chronobiol. Int..

[48] Rodriguez C., Mayo J.C., Sainz R.M., Herrera F., Antol I. (2004). Regulation of antioxidant enzymes: A significant role for melatonin. J. Pineal Res..

[49] Boutilier R.G., Donohoe P.H., Tattersall G., West T.G. (1997). Hypometabolic homeostasis in overwintering aquatic amphibians. J. Exp. Biol..

[50] Tattersall G.J., Ultsch G.R. (2008). Physiological Ecology of Aquatic Overwintering in Ranid Frogs. Biol. Rev..

[51] Holenweg A.K., Reyer H.U. (2000). Hibernation behavior of *Rana lessonae* and *R. esculenta* in their natural habitat. Oecologia.

[52] Milsom W.K., Jackson D.C. (2011). Hibernation and Gas Exchange. Compr. Physiol..

[53] Stinner J., Zarlinga N., Orcutt S. (1994). Overwintering behavior of adult bullfrogs, *Rana catesbeiana*, in northeastern Ohio. Ohio J. Sci..

[54] Costanzo J.P., Lee R.E. (2013). Avoidance and tolerance of freezing in ectothermic vertebrates. J. Exp. Biol..

[55] Kiss A.J., Muir T.J., Lee J.R.E., Costanzo J.P. (2011). Seasonal Variation in the Hepatoproteome of the Dehydration- and Freeze-Tolerant Wood Frog, *Rana sylvatica*. Int. J. Mol. Sci..

[56] Chan H.-K., Shoemaker K.T., Karraker N.E. (2014). Demography of Quasipaa frogs in China reveals high vulnerability to widespread harvest pressure. Biol. Conserv..

[57] Yu Y., Hu Y., Zhang Q., Zheng R., Shen B., Kong S., Li K. (2020). Female Preferences for Call Properties of Giant Spiny Frog (*Quasipaa spinosa*). Pak. J. Zool..

[58] Zheng R.-Q., Liu C.-T. (2010). Giant spiny-frog (*Paa spinosa*) from different populations differ in thermal preference but not in thermal tolerance. Aquat. Ecol..

[59] Schmittgen T.D., Livak K.J. (2008). Analyzing real-time PCR data by the comparative *C*_T_ method. Nat. Protoc..

[60] Hughes M.E., HogenEsch J.B., Kornacker K. (2010). JTK_CYCLE: An Efficient Nonparametric Algorithm for Detecting Rhythmic Components in Genome-Scale Data Sets. J. Biol. Rhythm..

[61] Lamia K.A., Papp S.J., Yu R.T., Barish G.D., Uhlenhaut H., Jonker J., Downes M., Evans R.M. (2011). Cryptochromes mediate rhythmic repression of the glucocorticoid receptor. Nature.

[62] Serino I., D’Istria M., Monteleone P. (1993). A comparative study of melatonin production in the retina, pineal gland and *Harderian gland* of *Bufo viridis* and *Rana esculenta*. Comp. Biochem. Physiol. Part C Pharmacol. Toxicol. Endocrinol..

[63] Neufeld-Cohen A., Robles M.S., Aviram R., Manella G., Adamovich Y., Ladeuix B., Nir D., Rousso-Noori L., Kuperman Y., Golik M. (2016). Circadian control of oscillations in mitochondrial rate-limiting enzymes and nutrient utilization by PERIOD proteins. Proc. Natl. Acad. Sci. USA.

[64] Lei X.G., Zhu J.-H., Cheng W.-H., Bao Y., Ho Y.-S., Reddi A.R., Holmgren A., Arnér E. (2016). Paradoxical Roles of Antioxidant Enzymes: Basic Mechanisms and Health Implications. Physiol. Rev..

[65] Reinke H., Asher G. (2019). Crosstalk between metabolism and circadian clocks. Nat. Rev. Mol. Cell Biol..

[66] Hardeland R., Coto-Montes A., Poeggeler B. (2003). Circadian rhythms, oxidative stress, and antioxidative defense mechanisms. Chronobiol. Int..

[67] Ruby N.F., Dark J., Burns D.E., Heller H.C., Zucker I. (2002). The Suprachiasmatic Nucleus Is Essential for Circadian Body Temperature Rhythms in Hibernating Ground Squirrels. J. Neurosci..

[68] Williams C.T., Barnes B.M., Richter M., Buck C.L. (2012). Hibernation and Circadian Rhythms of Body Temperature in Free-Living Arctic Ground Squirrels. Physiol. Biochem. Zool..

[69] Williams C.T., Radonich M., Barnes B.M., Buck C.L. (2017). Seasonal loss and resumption of circadian rhythms in hibernating arctic ground squirrels. J. Comp. Physiol. B.

[70] Florant G.L., Rivera M.L., Lawrence A.K., Tamarkin L. (1984). Plasma melatonin concentrations in hibernating marmots: Absence of a plasma melatonin rhythm. Am. J. Physiol. Integr. Comp. Physiol..

[71] Delgado M., Vivien-Roels B. (1989). Effect of environmental temperature and photoperiod on the melatonin levels in the pineal, lateral eye, and plasma of the frog, *Rana perezi*: Importance of ocular melatonin. Gen. Comp. Endocrinol..

[72] Chong N.W., Bernard M., Klein D.C. (2000). Characterization of the Chicken Serotonin N-Acetyltransferase Gene: Activation via Clock gene heterodimer/E box interaction. J. Biol. Chem..

[73] Son G.H., Chung S., Choe H.K., Kim H.-D., Baik S.-M., Lee H., Lee H.-W., Choi S., Sun W., Kim H. (2008). Adrenal peripheral clock controls the autonomous circadian rhythm of glucocorticoid by causing rhythmic steroid production. Proc. Natl. Acad. Sci. USA.

[74] Silveira E.J.D., Filho C.H.V.N., Yujra V.Q., Webber L.P., Castilho R.M., Squarize C.H. (2020). BMAL1 Modulates Epidermal Healing in a Process Involving the Antioxidative Defense Mechanism. Int. J. Mol. Sci..

[75] Chhunchha B., Kubo E., Singh D.P. (2020). Clock Protein Bmal1 and Nrf2 Cooperatively Control Aging or Oxidative Response and Redox Homeostasis by Regulating Rhythmic Expression of Prdx6. Cells.

[76] Niu Y., Zhang X., Zhang H., Xu T., Zhu L., Storey K.B., Chen Q. (2021). Metabolic responses of plasma to extreme environments in overwintering Tibetan frogs *Nanorana parkeri*: A metabolome integrated analysis. Front. Zool..

[77] Niu Y., Cao W., Zhao Y., Zhai H., Zhao Y., Tang X., Chen Q. (2018). The levels of oxidative stress and antioxidant capacity in hibernating Nanorana parkeri. Comp. Biochem. Physiol. Part A Mol. Integr. Physiol..

[78] Niu Y., Cao W., Storey K.B., He J., Wang J., Zhang T., Tang X., Chen Q. (2020). Metabolic characteristics of overwintering by the high-altitude dwelling Xizang plateau frog, *Nanorana parkeri*. J. Comp. Physiol. B.

[79] Niu Y., Chen Q., Storey K.B., Teng L., Li X., Xu T., Zhang H. (2022). Physiological Ecology of Winter Hibernation by the High-Altitude Frog *Nanorana parkeri*. Physiol. Biochem. Zool..

[80] Petrović V.M., Miliič B., Spasic M., Saičić Z. (1986). Copper-Zinc Containing and Manganese Containing Superoxide Dismutase in the Ground Squirrel/Citellus Citellus/—THE Effect of Hibernation. Free Radic. Res. Commun..

[81] Gattoni G., Bernocchi G. (2019). Calcium-Binding Proteins in the Nervous System during Hibernation: Neuroprotective Strategies in Hypometabolic Conditions?. Int. J. Mol. Sci..

[82] Bagnyukova T., Storey K., Lushchak V. (2003). Induction of oxidative stress in *Rana ridibunda* during recovery from winter hibernation. J. Therm. Biol..

[83] Buzadžić B., Blagojević D., Korać B., Saicic Z., Spasić M., Petrović V. (1997). Seasonal Variation in the Antioxidant Defense System of the Brain of the Ground Squirrel (*Citellus citellus*) and Response to Low Temperature Compared with Rat. Comp. Biochem. Physiol. Part C Pharmacol. Toxicol. Endocrinol..

[84] Mattice J.J.L. (2018). Regulation of Glutathione-Based Antioxidant Defenses in Response to Dehydration Stress in the African Clawed Frog, Xenopus laevis. Ph.D. Thesis.

[85] Mauvoisin D. (2019). Circadian rhythms and proteomics: It’s all about posttranslational modifications!. WIREs Syst. Biol. Med..

