# Supplementary material for: Hibernation with Rhythmicity in the Retina, Brain, and Plasma but Not in the Liver of Hibernating Giant Spiny Frogs (Quasipaa spinosa)
Source: Biology (Basel). 2022 May 9;11(5):722. doi: 10.3390/biology11050722 (PMC9138901; doi:10.3390/biology11050722)
Supplement: Supplementary file 1 [file biology-11-00722-s001.zip › biology-1674652-supplementary.pdf]

Table S1. Primer sequences used for cloning genes and for qPCR analysis.

Gene Name	Degenerated primer for cloning (5'→3')	Specific primer for qPCR (5'→3')
Clock	F:ACAARTCKGAGAAGAAGCGWAG	F: CAATGCCAGGAAGATGGATAAAT
	R: AWGGTAAGTGTTCAAGTAARGR	R: TGCGCAGGAAGTCAATGC
Bmal1	F: GGCTGGAYGAAGAYAACG	F: GCCGAGTGCCATAGACAAGTGT
	R: DGTGTTSGTGGAGACRAT	R: AGGGTGATGAAGGAACCATCTTTGAT
Cry1	F: GAYGAYCACGATGAGAAAT	F: TGCCAACTCCCTTCTTGCA
	R: GCTGCRGTGTARAAAAAYT	R: GGCATCCAAACCGCAGATAA
Per2	F:WSMRGARRYWGAWRAGGAS	F: TCACTGCAACCTGGAATTCAAG
	R:KCCAATGATRAAKGAMACY	R: TGGACAGGCTGCATAAGAAGCT
RORα	F:TGYGARGGYTGYAAGGGYTT	F:TGCAGAAACATCGCATGCA
	R:CTGCCABRTHAKCTGCTGDAG	R:TGCCTCCCCTGGTTGCT
AA-NAT	F: VGAGGAYGCBRTCAGYGT	F: CAACAAGGCAAGGGTTCCA
	R: SAGGAARTCYTCRCACAT	R: CAGACAGCGCAGGTATTGCA
Mel-1c	F: GTRWYGCMGAYYTBGTWGT	F: CGGCCAAAGGAACAAGAAAA
	R: AAMAGCCAYTCTGGAATSTK	R: TGCCCAAACAGTCAGTTCTTCA
SOD1	F: GAAGGCRATCTGTGTRYT	F: TCCACACTTTAACCCCTACAACAA
	R:CCAGTYAYCWKGCTCTCATT	R: CAACATGCCTCTCCTGGTCAT
SOD2	F: CTGACYTVMCWTATGAYTATGG	F:AACCTGAATATTGCAGAGGAGAATAC
	R: AGACCTGTKGTYCCTTGYAR	R: GCAATCTGAGCTGTAACATCTCCTT
CAT	F:CCGTCATTCATCCACTCTC	F: TGCGCCCGGAGTGTCT
	R:GTAGAAGGWCCAGGAAGGA	R: TGGGATACCACGGTCTGAGAA
GPx	F:YAACCARTTCGGRCACCA	F: GGCGTTCCCGATCTTTGAG
	R:RAAGTTCCAGGMGAYGTC	R: GGTGAAAAGCGGCAATTCC
β-actin	F:GGTCGCCCAAGACATCAG	F: GAGCAGGACTGGGTGTTCTTCA
	R:GCATACAGGGACAACACA	R: GCATTGTAACCAACTGGGACGA

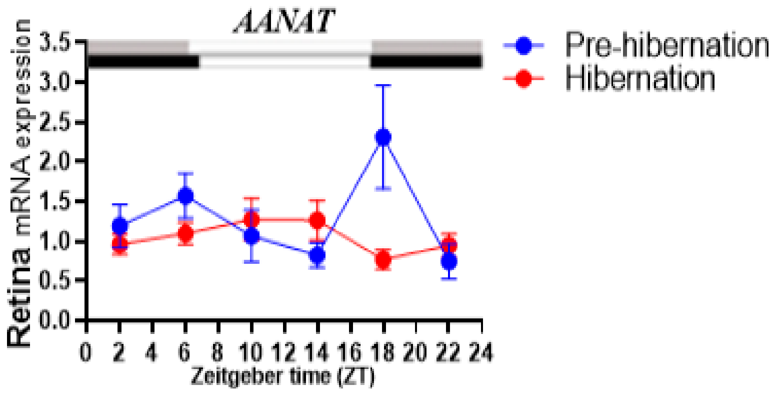

Figure S1. Comparison of mRNA expression of Retinal AANAT in the giant spiny frog (*Quasipaa spinosa*) between pre-hibernation and during hibernation. Rhythms are deemed significant when the adjusted p-value (ADJ.P) is <0.05. The times of sunrise and sunset on the sampling days, which were used to graph the daytime (open bar) and nighttime (gray bar for pre-hibernation and black bar for hibernation), were available from the local meteorological observatory.
